# Characterization of duck enteritis virus UL53 gene and glycoprotein K

**DOI:** 10.1186/1743-422X-8-235

**Published:** 2011-05-17

**Authors:** Shunchuan Zhang, Jun Xiang, Anchun Cheng, Mingshu Wang, Ying Wu, Xiaoyuan Yang, Dekang Zhu, Renyong Jia, Qihui Luo, Zhengli Chen, Xiaoyue Chen

**Affiliations:** 1 Institute of Preventive Veterinary Medicine, Sichuan Agricultural University, Wenjiang, Chengdu city, Sichuan, 611130, P.R.China; 2 Avian Disease Research Center, College of Veterinary Medicine of Sichuan Agricultural University, 46# Xinkang Road, Ya'an, Sichuan 625014, P.R. China; 3 Key Laboratory of Animal Disease and Human Health of Sichuan Province, Sichuan Agricultural University, Wenjiang, Chengdu city, Sichuan, 611130, P.R.China

## Abstract

**Background:**

Most of the previous research work had focused on the epidemiology and prevention of duck enteritis virus (DEV). Whilst with the development of protocols in molecular biology, nowadays more and more information about the genes of DEV was reported. But little information about DEV UL53 gene and glycoprotein K(gK) was known except our reported data.

**Results:**

In our paper, the fluorescent quantitative real-time PCR(FQ-RT-PCR) assay and nucleic acid inhibition test were used to study the transcription characteristic of the DEV UL53 gene. Except detecting the mRNA of DEV UL53 gene, the product gK encoded by UL53 gene was detected through the expression kinetics of UL53 gene by the purified rabbit anti-UL53 protein polyclonal antibodies. Western-blotting and indirect immunofluorescence assays were used to detect gK. From the results of these experiments, the UL53 gene and gK were respectively identified as a late gene and a really late protein. On the other hand, the indirect immunofluorescence assay provided another information that the intracellular localization of DEV gK was mainly distributed in cytoplasm.

**Conclusions:**

By way of conclusions, we conceded that DEV UL53 gene is a really late gene, which is coincident with properties of UL53 homologs from other herpesvirus, such as ILTV(Infectious Laryngotracheitis virus) and HSV-1(Herpes simplex virus type 1). The properties of intracellular localization about gK protein provided a foundation for further functional analysis and further studies will be focused on constructing of the UL53 gene DEV mutant.

## Background

Duck enteritis virus (DEV) is an *alphaherpesvirinae *that causes an acute, contagious and highly lethal disease in all ages of birds from the order *Anseriformes *(ducks, geese, and swans) [1-4]. DEV leads to heavy economic losses to the commercial duck industry due to its high mortality rate and decreased duck egg production [1].

Whilst most of the previous research work had focused on the epidemiology and prevention of this disease [5,6]. With the development of protocols in molecular biology, nowadays more and more information about the genes of DEV was reported, such as UL5 [7], gC [8-10], UL24 [11-13], UL31 [14,15], UL35 [16,17], UL46 [18], UL38 [19], gE [20], UL51 [21], TK gene [22] and so on. While no information about DEV UL53 gene was known except our reported data [23,24], UL53 gene encoded gK, one of DEV glycoproteins localized in the virion envelope, which played a major role in virus entry by mediating attachment of virions to cell-surface receptors and fusion of the viral envelope with the plasma membrane during penetration according to UL53 homologenes of other alphaherpesvirinae [25,26]. In order to investigate the roles that UL53 gene played in DEV replication and detect characterization of intracellular localization of DEV gK that was the product of UL53 gene, we carried out the fluorescent quantitative real-time PCR (FQ-RT-PCR) method, nucleic acid inhibition test and expression phase study to analyze the gene category of DEV UL53 and intracellular localization of DEV gK.

To begin dealing with the research project on the properties or functions of DEV UL53 gene and gK, we constructed the pET32b/UL53 plasmid and pMD18-T/β-actin plasmid, used the raised anti-DEV gK serum that specificly recognized the gK protein and revealed its temporal transcription course and intracellular localization in DEV-infected DEF cells. The research will provide useful data for DEV UL53 gene's properties or gK functional analysis, and also will be useful for further understanding the localization properties of alphaherpesvirus UL53 homologs.

## Results

### Fluorescent quantitative real-time PCR (FQ-RT-PCR) detect the UL53 gene transcript during DEV replication

#### Detect the specificity of the primers and the integrality or purity of the total RNA of each sample

**Figure 1:**
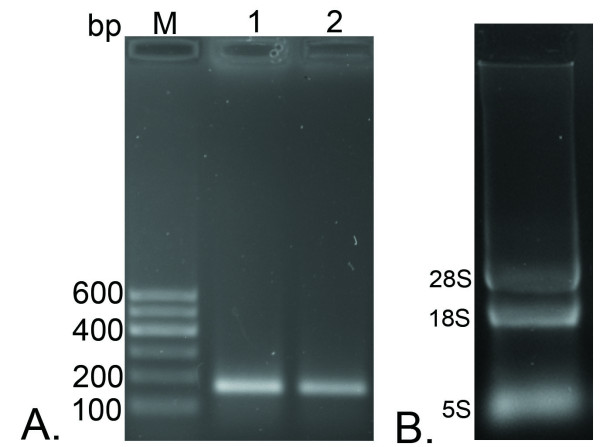
**Specific detection of the primers and integrality analysis of RNA samples**. A. Specific detection of the primers for DEV UL53 gene and the endogenous control β-actin gene. M, DNA marker--Marker I; 1, the specific amplification of the primers for DEV UL53 gene; 2, the specific amplification of the primers for the endogenous control β-actin gene. B. The integrality of RNA samples tested by agarose gel electrophoresis. Total RNA was isolated three bands(28S, 18S and 5S).

The primers P1,P2 for amplifying 164 bp of UL53 gene and primers P3,P4 for amplifying 178 bp of β-actin gene were detected the specificity by traditional PCR. The PCR products were fractionated on 1.5% agarose gel electrophoresis and stained with golden view. From the result (Figure 1A), the two pairs of primers had good specificity and no primer dimmer. The amplified products were the same with the predicted size.

The total RNA was extracted from mock or DEV-infected cells at given times using Trizol Reagent (Tiangen Biotech). After removing DNA in the RNA, the total RNA integrality analysis of each sample was detected by the agarose gel electrophoresis. From the figure 1B, the bands of 28S, 18S and 5S were clearly seen. At the same time, the purity of RNA was detected by nucleic acid-protein detecting instrument (Bio-Rad). The value of OD_260_/OD_280 _fluctuated between 1.8 and 2.0. Both results indicated the total RNA had good integrality and high purity.

#### Construction the double standard curves of FQ-RT-PCR

**Figure 2:**
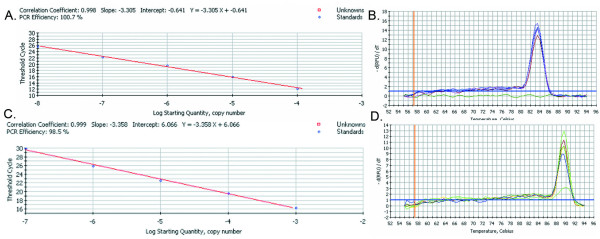
**The standard curves and melt curves of DEV UL53 gene and β-actin gene by FQ-RT-PCR**. A: Standard curve of DEV UL53 gene. The x-axis represented ten-fold dilutions of pET32b/UL53 plasmids, and the y-axis represented corresponding cycle threshold (Ct) values. The standard curve equation is Y = -3.305X-0.641. The correlation coefficient and the slope value of the regression curve were 0.998 and -3.305, respectively; B: Melt curve of DEV UL53 gene. There was only a singlet; C: Standard curve of β-actin gene. The x-axis represented ten-fold dilutions of pMD18-T/β-actin plasmids, and the y-axis represented corresponding cycle threshold (Ct) values. The standard curve equation is Y = -3.358X+6.066. The correlation coefficient and the slope value of the regression curve were 0.999 and -3.358, respectively; D: Amplification curve of β-actin gene. There was only a singlet, too.

The pET32b/UL53 plasmid was used to establish one of the standard curves of FQ-RT-PCR. Firstly, we diluted the initiate concentration of the pET32b/UL53 plasmid in order to contain the scope of cDNA sample products by the whole concentration gradients. Moreover, the appropriate diluted plasmids used as standard preparations were ten-fold dilution from 10^-1 ^to 10^-5^. According to the results of FQ-RT-PCR(Figure 2A), the standard curve equation was Y = -3.305X-0.641, the UL53 gene amplification efficiency was 100.7% and the correlation coefficient was 0.998. It's also clear that the crest value of melt curves is sole about 83.5°C(Figure 2B). Meanwhile the pMD18-T/β-actin plasmid was used to establish the other standard curve of FQ-RT-PCR. Using the same way to dilute the initiate concentration of the pMD18-T/β-actin plasmid, the result indicated that the β-actin gene amplification efficiency is 98.5%, the correlation coefficient is 0.999 (Figure 2C) and the crest value of melt curves is also sole about 89.5°C(Figure 2D). The standard curve equation of β-actin gene is Y = -3.358X+6.066.

#### Transcription analysis of DEV UL53 gene in DEF detecting by FQ-RT-PCR

**Figure 3:**
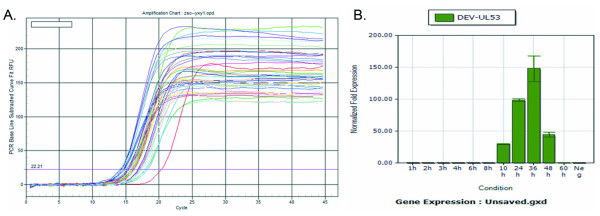
**FQ-RT-PCR was used to detect the transcript of UL53 gene**. A. The amplification curves of DEV UL53 gene and β-actin gene to detect the samples by FQ-RT-PCR. B. Gene expression analysis of UL53 gene by iQ™5 Optical System Software Version 2.1. The x-axis represented the conditions of different infected time, and the y-axis represented UL53 gene relative expressing quantity.

The isolated and processed RNA at given times was reverse transcribed into cDNA by Quantscript RT Kit(TIANGEN BIOTECH CO., LTD.). The quantitive primers(P1, P2) of DEV UL53 gene and primers(P3, P4) of the endogenous control gene β-actin were used to respectively detect the reverse transcribed sample, cDNA by FQ-RT-PCR. The fluorescent quantitation amplification curves of DEV UL53 gene and endogenous control gene β-actin were shown in figure 3A. In accordance with the endogenous control gene β-actin, the method of delta-delta Ct was used to analyze the relative transcripted quantity of DEV UL53 gene, and the data were calculated by iQ™5 Optical System Software Version 2.1. From the calculated result(Figure 3B), it's manifest that DEV UL53 gene begins to transcribe in DEF at 10 h.p.i.

### Detection of the UL53 gene transcript during DEV replication in the presence of a specific inhibitor of herpesvirus DNA polymerase

**Figure 4:**
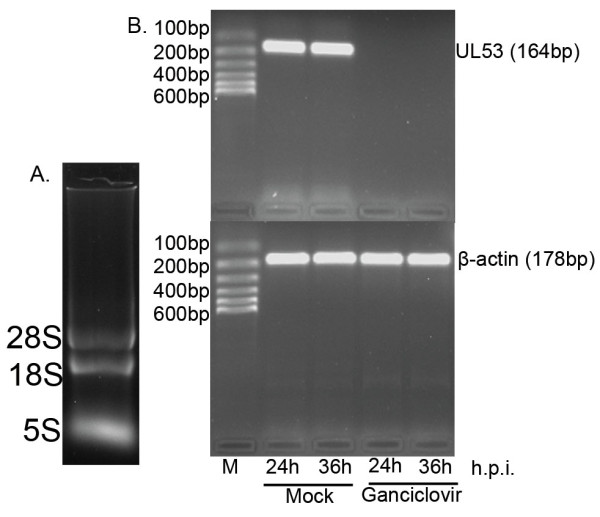
**Transcript detection of the UL53 by nucleic acid inhibition test**. A. The integrality of RNA samples tested by agarose gel electrophoresis. Total RNA was isolated three bands(28S, 18S and 5S). B. Detection of the UL53 transcript by nucleic acid inhibition test. M: DNA marker--Marker I. DEF were infected with DEV in the absence (Mock) or presence of 300 μg/ml ganciclovir and total RNA of infected cells were harvested at 24 h and 36 h postinfection followed by RT-PCR detection using specific primers to UL53 gene and β-actin gene respectively.

To characterize the temporal class of UL53 gene expression, DEF were infected with DEV in the presence or absence of ganciclovir. The total RNA was separately isolated from the mock and the test specimen that adding with ganciclovir, the specific inhibitor of herpesvirus DNA polymerase at different times(24 h, 36 h). The isolated RNA was processed with to remove the DNA within the sample RNA, detected the integrality of RNA by 1.5% agarose gel electrophoresis. From the figure 4A, it's obviously seen that the integrality of RNA is pretty good. RT-PCR analysis of total RNA harvested at different times by the primers(P1, P2) of DEV UL53 gene revealed a specific PCR product(164 bp) which was detected at 24 h.p.i and persisted in the subsequent infection stages(36 h) in the absence of ganciclovir, whereas no product was detected in infected cells in the presence of ganciclovir(Figure 4B). In contrast, the endogenous control gene β-actin always had a specific PCR product(178 bp) in the absence or presence of ganciclovir(Figure 4B).

### Characterization analysis of DEV UL53 gene and gene product gK

#### Western-blotting assay to study the expression kinetics of UL53 gene

**Figure 5:**
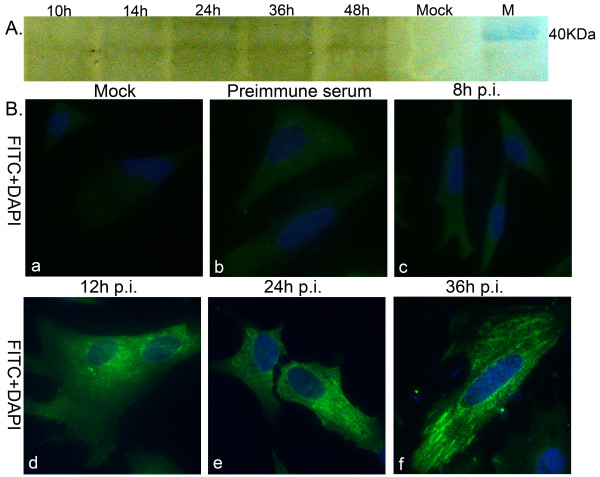
**Expression kinetics of DEV UL53 gene and intracellular location of the DEV gK in infected DEF by IIF**. A. Proteins isolated from mock or DEV-infected cells at different times (10 h.p.i. to 48 h.p.i.) were analyzed by Western blot. The specific band demonstrated the expected position for DEV gK (about 38 kDa). M represented standard protein molecular weight markers. B. Expression kinetics and intracellular location of the DEV gK in infected DEF. Mock-infected(a) and DEV infected DEF(b to f) were fixed as described in Methods. The samples were incubated with the purified rabbit anti-UL53 protein polyclonal antibodies(a, c to f) or preimmune serum(b), and reacted with fluorescein isothiocyanate (FITC)-conjugated secondary antibody, and then counter-stained with DAPI (the cell nuclei was stained with blue). The merged fluorescence microscopy images of DEF are shown from a to f with high magnification (400 ×).

In order to study the expression kinetics of UL53 gene, DEF cells were infected with DEV. Cell lysates were harvested at 10 h, 14 h, 24 h, 36 h and 48 h postinfection (p.i). Equal amounts of cell lysates were resolved by SDS-PAGE, and proteins in the gel were electrophoretically transferred to PVDF membrane and subjected to Western blot analysis with the purified rabbit anti-UL53 protein polyclonal antibodies. As seen in Figure 5A, DEV gK (molecular mass, approximately 37.7 kDa), was firstly detected in DEF cells till 14 h.p.i. The UL53 gene expression increased over time, reached a maximum at approximately 36 h.p.i and decreased in 48 h.p.i; this finding showed that gK is expressed in the viral replication cycle.

#### Indirect immunofluorescence assay(IIF) to detect the expression kinetics of UL53 gene and characterization of intracellular localization of DEV gK

The intracellular localization of gK protein was examined by indirect immunofluorescence staining. DEF were cultivated on coverslips and infected with DEV. The cells were processed at 8 h, 12 h, 24 h, and 36 h.p.i., and gK was detected by using the purified rabbit anti-UL53 protein polyclonal antibodies and fluorescein isothiocyanate (FITC)-conjugated secondary antibody. As can be seen from the Figure 5B, the gK mainly distributed in the cytoplasmic of infected DEF at 12 h.p.i. At the same time, the specific fluorescence increased over time of DEV infection(Figure 5B).

## Discussions

The expression of *Herpesviridae *genes is temporally controlled and coordinated in a cascade fashion [27]. In order to affirm the transcription characteristics of the DEV UL53 gene, the fluorescent quantitative real-time PCR(FQ-RT-PCR) assay and nucleic acid inhibition test were used to detect the mRNA transcribed by DEV UL53 gene. The technology of fluorescent quantitative real-time PCR is divided into relative quantitation method and absolute quantitation method according to final distinct data. In our paper, the relative quantitation method(delta-delta Ct) was used to analyze the transcription characteristics. But this method was based on the coincidental amplification efficiency between DEV UL53 gene and the endogenous control gene β-actin that was used as standardization of the other gene's expressing research. Before the beginning of relative quantitation method, the primers of the target gene DEV UL53 and endogenous control gene β-actin were specific and no primer dimmer through the PCR, which was also improved by melting curves of two pairs of primers. In preliminary experiment, it's necessary to respectively establish standard curves of DEV UL53 gene and β-actin gene and the Rotor-gene software would automatically generate the value of slope, amplification efficiency, correlation coefficient. If the slope and the correlation coefficient of the two standard curves are close to the theoretical values of -3.322 and 1.000, respectively, the consequence demonstrated the amplification efficiency of two genes was really approximate and the relative quantitation method of delta-delta Ct could be applied to analyze the transcription characteristics [28,29]. Otherwise this method could not be used. In our research, the difference of two standard curves slope between the target DEV UL53 gene and β-actin gene was not notable, therefore this method was valid. From the consequence of FQ-RT-PCR, we could clearly see that the DEV UL53 gene is firstly transcribed in 10 h.p.i.. According to the previous research, two identified immediate early products, namely protein kinase pUS3 and dUTPase, were firstly detected at 2 h.p.i. and 4 h.p.i., respectively [30,31]. In contrary, two another identified late products, namely tegument protein pUL31 and pUL51 were first detected at 6 h.p.i. and 8 h.p.i., respectively [15,32]. Hence, we concluded that UL53 was a late gene because the mRNA of UL53 could be firstly detected in 10 h.p.i.

On the background of gene category theory, gene temporal regulation condition should be analyzed when the infected cell was dealt with some canonical medicine [33]. Such as γ (late) gene could not be detected in the cell dealt with phosphonoacetate(DNA synthesis inhibition factor); α (immediate-early) gene can be detected in the cell dealt with protein synthesis inhibitor; β (early) gene can be inhibited by cycloheximide (protein synthesis inhibitor) while can't be inhibited by phosphonoacetate [34]. In nucleic acid inhibition test, no specific PCR product was detected in infected cells in the presence of ganciclovir(the specific inhibitor of herpesvirus DNA polymerase). So the DEV UL53 gene encoded a late transcript.

Except detecting the mRNA of DEV UL53 gene, the product gK encoded by UL53 gene was detected by the purified rabbit anti-UL53 protein polyclonal antibodies through the expression kinetics of UL53 gene. Western-blotting and indirect immunofluorescence assays were used to detect gK. From the results of both assays, the gK was respectively detected in 14 and 12 h.p.i. According to the recent reports of Wei Xie, Chanjuan Shen and Jun Xiang, the gK was a really late product [19,31,32].

On the other hand, the indirect immunofluorescence assay provided another information about the intracellular localization of DEV gK protein. That was valuable and useful data because little information about the intracellular localization of the DEV gK is known. However, due to the difficulties in the experimental determination of protein's cellular localization, the methods of theoretical prediction on the known sequence are becoming more important [35]. The analysis of the protein intracellular localization predicted by computer indicated that the gK locate in cytoplasm with 1.0%, cytoplasmic membrane with 92.6% and extracellular with 3.2%[36]. But the results of our research showed that cytoplasm fluorescence first appeared in DEV-infected cells at 12 h.p.i. At later times of infection, the specific fluorescence predominantly in cytoplasm region was increased. That was different between the predicted consequence and our experimental result. We presumed the gK maybe locate in the Golgi apparatus and endoplasmic reticulum (ER) membranes.

## Conclusions

By way of conclusions, through the experiments of the fluorescent quantitative real-time PCR, nucleic acid inhibition and the expression kinetics of UL53 gene, we conceded that DEV UL53 gene is a really late gene, which is coincident with properties of herpesvirus UL53 homologs, such as ILTV(Infectious Laryngotracheitis virus) UL53 gene and HSV-1(Herpes simplex virus type 1) UL53 gene [37,38]. From the result of indirect immunofluorescence assay, the gK protein showed obviously cytoplasmic staining in infected DEF. These properties of the gK protein provided a foundation for further functional analysis and further studies will be focused on constructing of the UL53 gene DEV mutant.

## Methods

### Detection of the UL53 gene transcript during DEV replication by fluorescent quantitative real-time PCR (FQ-RT-PCR)

#### Detecting the primers specificity of fluorescent quantitative real-time PCR (FQ-RT-PCR)

According to the DEV-UL53 gene(GenBank accession no.: EU071035), the primers of fluorescent quantitative real-time PCR (FQ-RT-PCR) was designed by IQ^5^(Bio-Rad). The forward primer(P1) was 5'-CGTATTGTATCTGCGGCT-3' and the reverse primer(P2) was 5'-CGAGTGGGCGAAATGAAC-3', which amplified a 164-base pair (bp) fragment of DEV-UL53 gene. As for the endogenous control gene β-actin, the forward primer(P3) was 5'-CCGGGCATCGCTGACA-3' and reverse primer(P4) was 5'-GGATTCATCATACTCCTGCTTGCT-3', which amplified a 178-base pair (bp) fragment [33]. Both primers were synthesized by Ivitrogen.

Using the pET32b/UL53 and pMD18-T/β-actin as templates, the specificity of the primers P1, P2 and P3, P4 were detected respectively. The 25 μl PCR reaction system was as follows: PrimerSTAR(Premix) DNA polymerase (0.625 Units) 12.5 μl, template 1.5 μl, each primer (20 pmol) 0.5 μl and the sterile ultrapure water 10 μl. Meanwhile the PCR reaction conditions were: 95°C for 4 min, 30 cycles of 94°C for 1 min, 59°C for 1 min, 72°C for 2 min, and then a final extension at 72°C for 10 min. The PCR products were fractionated on 1.5% agarose gel electrophoresis and stained with golden view.

#### Construction the double standard curves of FQ-RT-PCR

The pET32b/UL53 plasmid and pMD18-T/β-actin plasmid were constructed by our laboratory, which were used to establish the standard curves of FQ-RT-PCR. Firstly, according to the initiate concentration of two various plasmids, we diluted the initiate concentration in order to contain the scope of cDNA sample products by the whole concentration gradients. Moreover, the appropriate diluted plasmids used as standard preparations were ten-fold dilution from 10^-1 ^to 10^-5^. Lastly, the FQ-RT-PCR assay was performed to build the standard curves by an iCycler iQ™ real-time PCR detection system (Bio Rad Lab., Hercules, CA, USA). The program of FQ-RT-PCR was as follows: 1 min at 95°C followed by 40 three-step cycles of 30 sec at 94°C, 30 sec at 60°C and 30 sec at 72°C. The reaction system was a 20 μl reaction mixture containing 9 μl of POWER High-Capacity cDNA Reverse Transcription Kits SYBR Green PCR master mix (Applied Biosystems), 0.5 μl of each primer, 1 μl of plasmid template and 9 μl of sterile ultrapure water [9]. Each reaction had three replicates. Homogeneity of products from each reaction was confirmed by melt curve analysis. Analysis of the real-time PCR data was carried out using the comparative ΔΔCt method [39].

#### Duck embryo fibroblasts(DEF) culture and total RNA extraction

Duck embryo fibroblasts (DEF) was cultured at 37°C with 5% CO_2 _in minimal essential medium(MEM) supplemented with 100 U/ml penicillin, 100 μg/ml streptomycin, and 10% fetal bovine serum (FBS). After infecting DEV, DEF either mock-infected or infected were harvested at 1, 2, 3, 4, 6, 8, 10, 24, 36, 48 and 60 h.p.i. Total RNA was isolated from mock or DEV-infected cells at given times according to the instructions of Trizol Reagent (Tiangen Biotech Co., LTD).

#### Remove DNA in the RNA, and detect the integrality and purity of RNA

DNase I (RNase free) was added 10 U into 20-50 μg total RNA in order to remove the DNA within the sample RNA [40]. The reaction system was in a 10 μl mixture including 1 μl DNase buffer, 1 μl DNase I (RNase free), 0.5 μl RNase inhibitors and 7.5 μl sample RNA. After mixing the mixture, the 10 μl mixture was digested in 37°C about 30 min.

In order to detect integrality of the RNA, 5 μl sample RNA was detected by 1.5% agarose gel electrophoresis. At the same time, the purity of RNA was detected by nucleic acid-protein detecting instrument (Bio-Rad).

#### Prepare the sample cDNA through reverse transcription and detect the sample cDNA by FQ-RT-PCR

Total RNA extracted from various time was reverse transcribed into cDNA by Quantscript RT Kit (TIANGEN BIOTECH CO., LTD.) and the products were reserved at -20°C. The reaction system of reverse transcription was in 20 μl mixture containing 2 μl 10 × RT mix, 2 μl dNTP, 2 μl Random primers, 1 μl Quant Reverse Transciptase, 11 μl RNase-free H_2_O and 2 μl RNA template. When mixing the mixture, the mixture was incubated for 60 min at 37°C for reverse transcription.

After the reverse transcription, the cDNA products from mock or DEV-infected cells at various times were detected by fluorescent quantitative real-time PCR assay. The primers(P1, P2) and (P3, P4) were used to amplify 164-base pair (bp) fragment of DEV UL53 gene and 178 bp fragment of the endogenous control gene β-actin, respectively. The reaction system and procedures were the same with construction of the double standard curves of FQ-RT-PCR. The fold change in expression of UL53 gene relative to the endogenous control gene (β-actin) at various time points was calculated as Fold change = Log(2^-ΔΔCt^), where ΔΔCt = (Ct,_Target_-Ct,_Reference_)_Time x_- (Ct, _Target_-Ct, _Reference_)_Time 0 _[9,33].

### Detection of the UL53 gene transcript during DEV replication in the presence of a specific inhibitor of herpesvirus DNA polymerase

#### Cell culture and sample preparation

Duck embryo fibroblasts (DEF) was cultured at 37°C with 5% CO_2 _in minimal essential medium(MEM) supplemented with 10% fetal bovine serum (FBS), 100 U/ml penicillin and 100 μg/ml streptomycin. When the DEF were grew into cell monolayer in 20 ml culture flask, we respectively added the ganciclovir (a specific inhibitor of herpesvirus DNA polymerase) with 300 μg/ml into the DEF infected with 150 μl DEV(TCID_50 _= 10^-6.334^/100 μl) [32,41]. In contrary, the mock was only infected with 150 μl DEV(TCID_50 _= 10^-6.334^/100 μl). We separately harvested three different samples at 24 h.p.i or 36 h.p.i. Each sample has three replications.

#### RNA extraction and cDNA preparation

After dumpaging supernatant, the remaining cells were used to extracted total RNA by Trizol Reagent (Tiangen Biotech) from various samples, which was carried out according to the manufacturer's instructions. Moreover, the reaction procedures and reaction conditions of removing DNA within the total RNA and implementing reverse transcription were the same with that of fluorescent quantitative real-time PCR (FQ-RT-PCR). And then, the products were storaged at -20°C for use.

#### Conventional PCR was used to detect the targeted fragment

Using the above products as templates, the primers(P1,P2) and primers(P3,P4) were respectively used to detect the UL53 gene and β-actin gene. The reaction system was carried out in a 25 μl reaction mixture containing 0.5 μl of each primer(20 pmol), 1.5 μl cDNA template, 12.5 μl PrimerSTAR(Premix) DNA polymerase (0.625 Units) and 10 μl water (all reagents were purchased from TaKaRa). The PCR conditions were: 95°C for 4 min, 30 cycles of 94°C for 1 min, 59°C for 1 min, 72°C for 2 min, and then a final extension at 72°C for 10 min. The PCR products were fractionated on 1.5% agarose gel electrophoresis and stained with golden view.

### Expression phase analysis of duck enteritis virus UL53 gene

#### Western-blotting assay to study the expression kinetics of UL53 gene

To study the expression kinetics of UL53 gene, DEF were either mock or infected with DEV as described above and harvested at 10, 14, 24, 36, and 48 h.p.i. Cell lysates were in SDS sample buffer, which were separated by SDS-PAGE, and simultaneously electrophoretic transfered to polyvinylidene difluoride (PVDF) membranes (Bio-Rad Lab., Hercus, CA, USA) at 120 V for 90 min [9,19]. Non-specific protein binding was blocked through dealing with PVDF membranes with TBST (20 mmol Tris-HCl, 150 mmol NaCl, pH 7.4, and 0.05% Tween-20) containing 3% bovine serum albumin (BSA). And then, the purified rabbit anti-UL53 protein polyclonal antibodies were used as primary antibody at a dilution of 1:50. The PVDF membranes were overnightly incubated at 4°C. After washing membranes three times with TBST, the goat anti-rabbit peroxidase-labeled antibody (KPL Inc., Gaithersburg, Maryland, USA) was used as secondary antibody to incubate the membranes. Lastly, the membranes were developed in diaminobenzidine (DAB) substrate buffer and photos were taken by a digital camera.

#### Indirect immunofluorescence assay(IIF) to detect the expression kinetics of UL53 gene and characterization of intracellular localization of DEV gK

DEF cells were cultivated on coverslips in six-well plates, which were mock or infected with DEV. The coverslips were harvested at different times (8, 12, 24, 36 h.p.i) and then fixed with 4% paraformaldehyde for 12 h at 4°C. The coverslips were washing three times with PBS, and then blocked the coverslips with PBS containing 3% BSA at 37°C for 1 h [9]. The purified rabbit anti-UL53 protein polyclonal antibodies (1:100 dilution) were used to incubate coverslips at 37°C for 2 h. After washing three times with PBS including 0.1% Tween-20, the coverslips were incubated with fluorescein isothiocyanate (FITC)-conjugated goat anti-rabbit IgG(1:150 dilution) (Sino-American Biotechnology Co., Shanghai, China) for 1 h at 37°C. At last 4,6-Diamidino-2-phenylindole (DAPI; Sigma) staining was used to visualize the cell nuclei [21]. Immunofluorescence photos were taken and recorded by the Bio-Rad MRC 1024 imaging system.

## Competing interests

The authors declare that they have no competing interests.

## Authors' contributions

SCZ, JX carried out most of the experiments and drafted the manuscript. ACC, MSW, YW, XYY, DKZ, RYJ, QHL, ZLC and XYC helped in experiments and drafted the manuscript.. All authors read and approved the final manuscript.
